# P347: Analysis of the supply and management of medicines in private hospitals

**DOI:** 10.1186/2047-2994-2-S1-P347

**Published:** 2013-06-20

**Authors:** B Antonio Carl

**Affiliations:** 1 International Polyclinic Hotel Dieu, Abidjan, Côte d'Ivoire

## Introduction

In Côte d'Ivoire, the private sector accounts for 40% of health care provision, but how these institutions, expanding obtain supplies of medicines and how they manage these drugs.

## Objectives

This is the first analysis to achieve pharmacies internal use private sector health and highlight the importance of pharmacy in private clinics.

## Methods

This is a covert investigation, visiting 37 private clinics legally constituted Organization 10 national non-profit, 8 clinics and 1 clinic illegally installed "Chinese" legally installed. The study took place from 01/09/2013 to 28/02/2013, a period of six months.

## Results

Almost all private clinics have a pharmacy internal use or a hundred. But according to the National Order of Pharmacists of Côte d'Ivoire there are only 5 hospital pharmacists in the private sector after adding a little investigation fifteen establishments that employs a part-time pharmacist. To this we see that there is no control. In this context how private clinics supply of drugs? First obstacle: the lack of dedicated wholesale distributor. It is very difficult to get clear answers. In this respect, our analysis is done in two stages, the Pharmacy for internal use with at least one pharmacist present and others. The presence of the pharmacist entails a radical change in the management of the pharmacy use internal traceability drug store the rules, proper stock management, production almost zero obsolete. In fact, the hospital pharmacist assumes responsibility under its function. As we proceeded quietly to perform visits to private health centers.

## Conclusion

The ministry of health and the fight against AIDS sounding the alarm, dated 02.07.2013, a note entitled "ALERT" said the invasion of counterfeit drugs in the whole country's health. To date there is only one text that defines a pharmacy for internal use, it is section 577 of the Public Health Code of 1956 French inherited from the colonial era.

## Disclosure of interest

None declared

